# Similarity Evaluation on the Compound TCM Formulation “Huoling Shengji Granule” and Its Placebo by Intelligent Sensory Evaluation Technologies and the Human Sensory Evaluation Method Based on Critical Quality Attributes

**DOI:** 10.1155/2021/6637326

**Published:** 2021-04-14

**Authors:** Mei Wu, Chengjie Guo, Ning Guo, Tianyi Zhang, Youjie Wang, Yuan Wang, Xiao Lin, Fei Wu, Yi Feng

**Affiliations:** ^1^Engineering Research Center of Modern Preparation Technology of Traditional Chinese Medicine, Ministry of Education, Shanghai University of Traditional Chinese Medicine, Shanghai, China; ^2^College of Chinese Materia Medica, Shanghai University of Traditional Chinese Medicine, Shanghai, China

## Abstract

To evaluate the similarity of Huoling Shengji granule (HLG) and its placebo at both granules and solution status, the innovative methods that consist of intelligent sensory evaluation technologies and human sensory evaluation methods were developed based on critical quality attributes (CQAs) of granule. The CQAs for traditional Chinese medicine (TCM) placebo granule were mainly divided into three categories: formulation attributes, visual attributes, and attributes of taste and smell. In this investigation, the novel intelligent sensory evaluation technologies including the physical property testing apparatus, computer vision system, color card, and electronic tongue (E-tongue) were employed for characterization of CQAs of HLG and its placebo. Meanwhile, human sensory evaluation by test panels was used to description the HLG and its placebo in terms of appearance, color, taste, and smell. On that basis, the similarity of placebo to CQAs of HLG was assessed by calculating the angle cosine values. The intelligent and human sensory evaluation results showed that the similarity values of HLG and its placebo about the CQAs at granule and solution status were all close to 1, which means that the two preparations have high similarities. In this study, the established similarity evaluation methods based on the CQAs were convenient and reliable, which can be utilized to evaluate the similarity of TCM granule and their placebo at granule and solution status, and demonstrated to be well applied in placebo-controlled trials.

## 1. Introduction

According to the recent surveys, from 2008 to 2018, the number of randomized placebo-clinical trials has always shown an upward trend [1]. Traditional Chinese medicine (TCM) compound formulations have gradually been the investigation of hot spots at the same time due to that TCM was characterized by safety and the comprehensive effect and as a complementary treatment to Western medicine [2]. Therefore, the TCM placebo may play a key role in evaluating the effectiveness of TCM compound formulations in clinical trials. Although the term “placebo” was first introduced into medical jargon and gradually applied controversially in clinical trials in the late eighteenth century, the studies of TCM placebo that meet the requirements of similarity are poor [3]. Moreover, evaluating and developing placebos for clinical trials is more challenging, particularly when the TCM compound formulations that contain rich color, special appearance, complex taste, and smell are different from the chemical drugs. It is difficult to simulate and evaluate the critical quality attributes (CQAs) for TCM compound formulations in terms of appearance, color, smell, and taste. The CQAs are not only the key properties of formulations, which affect the quality of preparations, but also the key parameters for evaluating preparation. For TCM placebo granule, the CQAs are mainly divided into three categories [4]: (i) formulation attributes (according to the different formulations and the quality control items, such as density, particle size, viscosity, dissolubility, fluidity, and texture properties), (ii) visual attributes (such as packaging, appearance, specifications, color, and clarity), and (iii) attributes of smell and taste (smell, taste, and irritation).

In this work, a TCM compound formulation named as “Huoling Shengji granule” (HLG) was developed, which was designed to treat amyotrophic lateral sclerosis (ALS) [5]. The clinical studies have shown that HLG could effectively prolong the lifespan and extend the disease duration in ALS patients. In order to further verify the ameliorative effect of HLG on ALS patients, a double-blind, placebo-controlled clinical trial would be conducted, while the placebo control implementation is an effective guarantee for blindness and clinical efficacy. Nevertheless, similarity evaluation is essential for the successful developing of TCM placebo before clinical trials. Hence, it is necessary to assess the similarity of CQAs for HLG and its placebo. Conventionally, the primary evaluation methods for the similarity assessment of TCM placebos are the human sensory evaluation methods. However, it is laborious and easy to be affected by the objective and subjective factors, thereby limiting its application in comparing similarity between HLG and its placebo [6]. In order to overcome the above disadvantages of human sensory evaluation methods, the development of intelligent sensory evaluation technologies based on accurate parameters to standardize objective similarity assessment is, thus, a significant unmet need in preparation and evaluation of TCM placebos. As reported by many studies, intelligent sensory evaluation technologies have been increasingly applied in food and pharmaceutical research studies [7–9], which include objective technologies that imitate the characteristics of human senses, such as senses of sight, smell, and taste, consisting of the computer vision (CV) system, electronic nose (E-nose), and electronic tongue (E-tongue) [10]. The CV system is a technology that breaks the limitations of traditional color analysis and can perform color analysis quickly [11]. E-nose and E-tongue have a similar working principle, in which the overall analysis of the sample is carried out through the signal collected by the sensor array, combined with the data analysis system, and they have been designed to simulate human smell and taste in the best way [12]. So, the CV system, E-nose, and E-tongue are described in the literature as the main methods for evaluating the organoleptic attributes of medicines [13–16]. Kiani et al. developed an integrated system based on the CV system and E-nose for determining adulteration of saffron [17, 18]. Preis et al. verified that maltodextrins, analyzed by E-tongue, could be a safe taste mask excipient, which, for example, can be applied in syrup development for pediatric use with good taste [19]. The human sensory evaluation method is the only method to accurately perceive different sensory intensity for pharmaceutical formulations from volunteers. As the old saying goes, there are a thousand Hamlets in a thousand people's eyes. As compared to the single human sensory evaluation method, intelligent sensory technologies that can avoid a certain degree of ambiguity and uncertainty from objective and subjective factors are to sense the different signals via the machine system. Thus, the research aims to focus on developing intelligent sensory technologies combined with human sensory evaluation methods that are suitable for evaluating similarity between HLG and its placebo in the form of granule and solution to assessment effectively. In regard of intelligent sensory technologies, formulation attributes were determined by physical property testing apparatus. Second, the visual attributes of the RGB color space, HSV space, and gray values were extracted by the computer vision system and color card to characterize visual information. And then, the taste attributes were determined by E-tongue. In addition, the study also adopted the human sensory evaluation method to evaluate the similarity of the two preparations.

Thus far, few studies have been systematically conducted to assess the similarity of TCM formulation and its placebo. Therefore, according to the advantages of organoleptic evaluation methods, this study aimed to develop the noninvasive, objective, and convenient intelligent sensory evaluation technologies, combined with human sensory evaluation methods, to evaluate the similarity of HLG and its placebo in different status (granule and solution). Furthermore, the novel way of combining digital and sensory analysis makes the preparation of HLG placebo more assured, similar, and can be used for reference, so as to increase the imitation accuracy and identify the critical steps in the placebo preparation process. Such methods could be used to monitor the sensory quality control of samples. It also lays the foundation for clinical trial research studies of TCM placebo [20].

## 2. Materials and Methods

### 2.1. Experimental Materials

In this research, tartrazine, sunset yellow, and *β*-carotene were acquired from Tianjin Duofuyuan Industrial Co., Ltd. Dextrin was obtained from Anhui Sunhere Pharmaceutical Excipients Co., Ltd. (Anhui, China). The TCM compound formulation of HLG extract powders was made from Radix Astragali (lot no. 180671), Fructus Corni (lot no. D180059), Radix Rehmanniae (lot no. 180629), *Poria cocos* (lot no. 1806093), and *Atractylodes macrocephala* Koidz (lot no. D1810058), all of which were purchased from Sichuan Neautus Traditional Chinese Medicine Co., Ltd. [20].

#### 2.1.1. Preparation of HLG

Herbs were weighed and mixed according to the proportion as prescribed (Epimedium herb : Radix Astragali : Fructus Corni : Radix Rehmanniae : *Poria cocos* : *Atractylodes macrocephala* Koidz = 5 : 6 : 4 : 5 : 3 : 3), and decocted for 1 h, followed by hot filtration with gauze. The filtrate was concentrated to the relative density of 1.25 or 1.38 by the rotary evaporator at −0.092 MPa and 60°C. Then, the concentrate was vacuum-dried with temperature 80°C to obtain extract powders [21]. The HLG extract powders and dextrin at a ratio of 3 : 7 (w/v) were performed dry granulation to acquire HLG granule.

#### 2.1.2. Preparation of HLG Placebo

Tartrazine, sunset yellow, *β*-carotene, and dextrin were performed by dry granulation to acquire HLG placebo samples.

### 2.2. Apparatus

The thin-layer chromatography (TLC) visualizer system with CCD lens, light source, and black box connected to computer with winCATS software installed for imaging recording was purchased from CAMAG of Switzerland. When samples were put into the TLC box, the image was recorded and saved. Then, the software MATLAB R2016b (MathWorks Inc., Natick, MA, USA) was used to image analysis for the computer vision system. The objective taste evaluation method was performed using an Astree II electronic tongue (Alpha M.O.S, Toulouse, France). The E-tongue consisted of a hexadecimal autosampler, Ag/AgCl reference electrode, a data acquisition system, an AlphaSoft V12 station, and seven sensors (ZZ-2808-12-535, AB-2808-11-552, GA-2808-12-415, BB-2808-12-327, CA-2808-12-447, DA-2808-11-438, and JE-2808-12-296, which were abbreviated into ZZ, AB, GA, BB, CA, DA, and JE, respectively). The seven sensors were specifically developed for taste detection. The E-tongue was connected to computer with the Astree II software (Alpha M.O.S) installed [22]. The key granule characteristics were determined using the powder comprehensive characteristic tester (BT-1000 model, Dandong Baite Instrument Co., Ltd., China), infrared moisture analyzer (HE53 model, Mettler Toledo, Switzerland), Malvern laser particle size analyzer (2000 model, Malvern Instruments Ltd., UK), densitometer (Densitometer DMA35N, Anton Paar, Australia), rotational viscometer (LVDV-I + Prime type, Brookfield, USA), and pH meter (Acidity meter PB-10 model, Sartorius Scientific Instruments Co., Ltd., Germany).

### 2.3. The Similarity Evaluation of Critical Quality Attributes

#### 2.3.1. The Formulation Attributes

*(1) Angle of Repose (AR)*. Angle of repose was used as an indicator of fluidity properties. The prepared granule was uniformly flowed through the funnel of a powder comprehensive characteristic tester (BT-1000 model, Dandong Baite Instrument Co., Ltd., China) to form a powder heap above the metal platform [23]. Then, the protractor was used to assist the determination of AR between cone slope and the plane from the three sides. The AR value presented was an average value of 3 measurements.

*(2) Moisture Determination*. About 2 g granule was immediately analyzed using an infrared moisture analyzer (HE53 model, Mettler Toledo, Switzerland) at 105°C. Duplicate tests were run for each sample. Therefore, the results were calculated to indicate the precision of the test [24].

*(3) The Median Particle Size*. Approximately 3 g granule was placed in the dry sampler of the Malvern laser particle size analyzer (2000 model, Malvern Instruments Ltd, UK) [25]. The equipment took air as a dispersion medium, and the parameters were adjusted with a refractive index of 1.5. It was used to measure the powder particle size distribution *D*_0.5_.

*(4) Solubility*. The solubility of granule was measured according to the Chinese national standard GB/T 5009.3-2015. The 10 g of the granule was stirred in hot water for 5 minutes, and the solubility was observed. Then, the solution was filtered, and the filter paper was dried for 4 h at 100 ± 5°C in a convection oven [26]. The samples were cooled in a desiccator and reweighed to constant weight (±2.0 mg). The solubility was estimated on filter paper weight for each sample.

*(5) Relative Density Measurement*. The solution was prepared at the target concentration and measured at 35°C using a densitometer (Densitometer DMA35 N, Anton Paar, Austria) in parallel three times [27].

*(6) Viscosity Measurement*. The viscosity of solution was measured using a rotational viscometer (LVDV-I + prime type, Brookfield, USA.) with rotal S1, 50 rpm, and 35°C.

*(7) Acid and Alkali Determination*. The solution was measured using a pH meter (Acidity meter PB-10 model, Sartorius Scientific Instruments Co., Ltd., Germany) with temperature of 35°C.

*(8) Clarity*. The clarity of solution was objectively characterized according to physical properties of solution such as viscosity, pH, density, and solubility.

#### 2.3.2. Statistical Processing

The angle cosine values were performed by the MATLAB software. They were used to characterize the similarity of HLG and its placebo about CQAs. All measurements were computed as follows:

(1) $$\begin{array}{c} \cos\left(\theta \right)=\frac{a\cdot b}{\left\|a\right\|\cdot \left\|b\right\|}. \end{array}$$

The values of *a* and *b*, which reflect the trend of the phase point in the phase space, can be obtained via MATLAB software, where *a* and *b* represent the corresponding points of the sensory attribute data of HLG and its placebo, respectively. The closer the value is to 1, the higher the similarity is [28].

### 2.4. The Visual Attributes

#### 2.4.1. Color Card Evaluation

The color difference between HLG and its placebo was evaluated using PANTONE International Standard Color Card (GP1601N, Pantone International Color Card Co., Ltd., USA) as a reference.

#### 2.4.2. Computer Vision System Evaluation

*(1) Preparation of Samples for Vision Evaluation*. Samples (HLG and placebo granule) were put into colorless transparent glassware, and images were acquired with the TLC visualizer imaging system.

Samples (HLG and placebo granule) were dissolved in purified water. A part of the solution was placed in a colorless transparent glassware, and images were acquired with the TLC visualizer imaging system.

*(2) Image Acquisition*. The study adopted the TLC Visualizer imaging system that was part of the photo module of the TLC thin layer spotter to acquire images. The Visualizer imaging system's CCD lens has a pixel size of up to one million and high resolution [10]. It used the high level white light as its light source in order to avoid disturbing from other light sources. The computer installed with WinCATS software was used for capturing and storing the images.

*(3) Image Visual Information Extraction*. The visual information of images with the same size 250 *∗* 250 pixels was digitally analyzed by the MATLAB 2016b software that collected the related visual information of each pixel RGB (red, green, and blue) color space, HSV (hue, saturation, and value), color space, and gray value. The mean, variance, and standard deviation of RGB and HSV information formed the angle cosine algorithm [29], which was described as the vision similarity of samples.

The original image was divided into foreground and background by using thresholding [30]. First, the original RGB image recorded was converted into LAB image, and then, a gay scale of RGB equivalent was generated and transformed into gray space. Finally, the MATLAB software had developed a function to calculate gray values by image pixel color difference.

#### 2.4.3. Statistical Processing

All experiments were replicated in three flasks, and the data were presented as the mean, variance, and standard error of three independent experiments. The RGB values, HSV values, and gray values were performed by the MATLAB. Finally, the similarity evaluation results of visual attributes were presented by the angle cosine values.

### 2.5. The Taste Attributes

#### 2.5.1. Electronic Tongue Measurement

The experiment aimed to compare the taste of HLG and its placebo by means of an E-tongue that comprised of 7 no specific sensors. The functionality of the sensors was proven by a conditioning, calibration, and diagnosis procedure performed before every measurement [31]. All the samples were analyzed by E-tongue for 120 s. The acquisition frequency was 1 time/second, and the stable sensor responses between 100 s and 120 s were transformed to an average value for statistical analysis [32].

Based on the electrochemical data collected by the sensor, the E-tongue with the AlphaSoft program (Alpha MOS) was submitted to principal component analysis (PCA) in order to assess the similarity of HLG and its placebo. When determining taste difference of samples, the PCA extracted the response of sensors for data conversion and dimensionality reduction, and then, the dimensionality reduced eigenvectors were linear classified. Finally, on the PCA chart, the abscissa represented the contribution rate (or weight) of the PC1, and the ordinate represented the contribution rate (or weight) of the PC2. The greater the contribution rate was, the better the principal component could reflect the sensor's response information. A discrimination index (DI), ranging from negative values to 100, is reported on a PCA map [33]. The higher the index number, the better the discrimination (or less similarity) between samples or groups shows. So, the DI was used to distinguish different samples.

#### 2.5.2. Investigation of Sample Concentration and Precision

To achieve better responses and differentiation of E-tongue, the solution of HLG was investigated at 10, 1, and 0.1 mg/mL. And then, the 0.1 mg/mL concentration was selected as the best discrimination ability. At the concentration level of 0.1 mg/mL of samples, the precision of the 7 sensors was better.

#### 2.5.3. Preparation of Samples for E-Tongue Evaluation

According to the prescription of HLG and its placebo, all samples including the excipient of HLG, HLG extract powders, HLG granule, 10% extract powders of HLG granule, taste excipient of placebo, free of taste excipient, and placebo granule were made into a concentration of 0.1 mg/mL. After being filtered, the 80 mL of solution was placed in a beaker and loaded into the autosampler tray.

### 2.6. Human Sensory Evaluation

The human sensory similarity of CQAs between HLG and its placebo was evaluated by a 6-number healthy volunteer panel (3 women, 3 men; age of 20–55 years) from Shanghai University of Traditional Chinese Medicine. The human sensory evaluation scores were the sum of formulation, visual, taste, and smell attributes scores. The mean scores of CQAs for every characteristic attribute were calculated and characterized by angle cosine values [34].

#### 2.6.1. Study Design

With the aim of ensuring the effectiveness of placebo control, 6 healthy volunteers (half males and half females) were asked to provide individual scores on the 0–4 scale (fully consistent with 4 points, completely inconsistent with 0 points, and the score could not be an integer) for the CQAs about the similarity degree between HLG and its placebo [35]. HLG and its placebo were completely randomly divided into drug A and drug B. HLG was used as the reference drug, and the similarity scores of drug A and drug B were compared with the reference drug. Similarity evaluation results were presented by the form of scores.

#### 2.6.2. Statistical Processing

The “*t*-test” for paired sample was conducted using SPSS 21.0 to determine continuous variables and inspection level *α* = 0.05. All the statistical analyses were performed using MATLAB 2016b to calculate the similarity of the two preparations. The angle cosine value was used as the final evaluation index, whose formula could refer to equation (1).

## 3. Results

### 3.1. Formulation Similarity Evaluation

**Figure 1:**
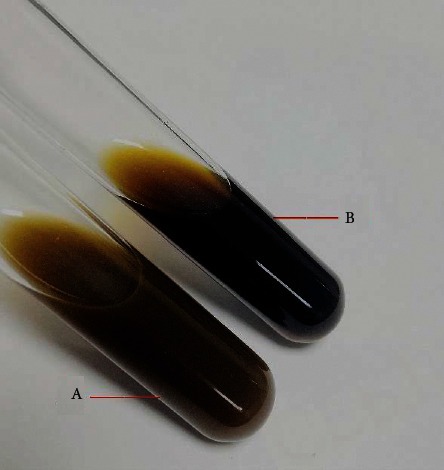
The solution of HLG and the placebo (A, HLG; B, HLG placebo).

The formulation attributes data of granule between HLG and placebo are given in Table 1. The granule similarities of the two preparations were objectively characterized by the angle cosine value, and the result was 0.9976. Comparing with the clarity of the solution between HLG and placebo by direct observation and objective analysis, the similarity of the solution between HLG and placebo was 0.9997 (Table 2 and Figure 1). This study intelligently characterized similarity degree about the clarity of the solution between the HLG granule and its placebo by the angle cosine value. The closer the angle cosine value is to 1, the better the similarity. As shown in Figure 1, the clarity of the two preparations was similar.

### 3.2. Similarity Evaluation of Visual Attributes

#### 3.2.1. Color Card Evaluation

According to Table 3, the color card numbers of the two preparations differed by only one, indicating their high similarity in color. The color of HLG and its placebo was objectified and standardized by color card, which was convenient for quick reference and evaluation [36]. As one of the most important evaluation indexes, visual information is often used for visual attributes of placebo, which is usually evaluated by trained specialists. However, above evaluation methods are easily affected by environment, illumination, subjective visual difference, and so forth. Therefore, the intelligent sensory evaluation technology-computer vision system was developed.

#### 3.2.2. Computer Vision System Evaluation

**Figure 2:**
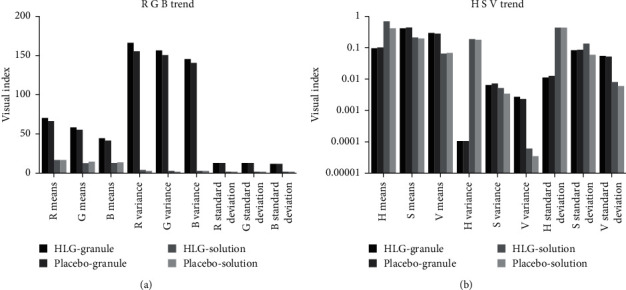
The results of RGB and HSV visual indicators between HLG and the placebo both at granule and solution status (R, red; G, green; B, blue; H, hue; S, saturation; V, luminance).

**Figure 3:**
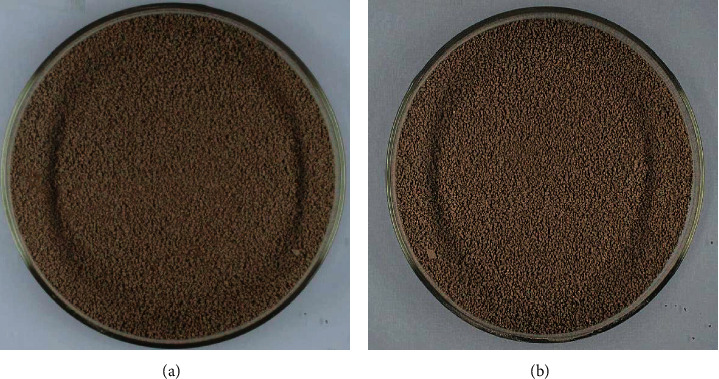
Color comparison of HLG and the placebo. (a) HLG. (b) The placebo.

**Figure 4:**
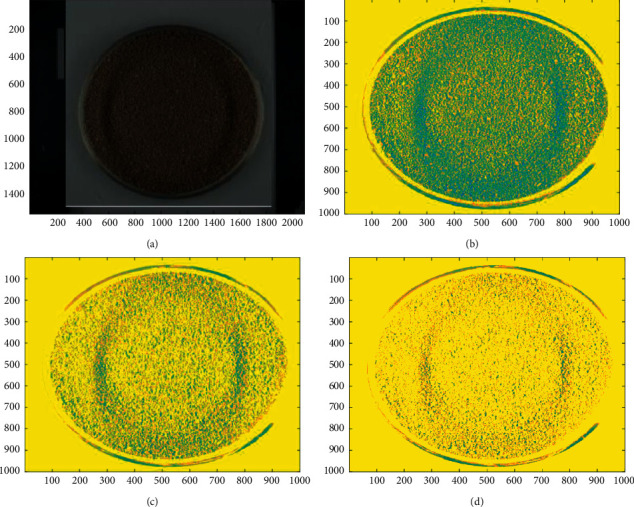
Grayscale image of HLG and the placebo. (a) Original image of HLG, (b) LAB image of the HLG segmentation, (c) grayscale diagram of HLG, and (d) grayscale image of HLG.

In this study, the mean, variance, and standard deviation of the RGB and HSV values were calculated by the MATLAB 2016b after the data were extracted from the image between HLG and the placebo (granule and solution). The RGB color space, HSV space, and image gray value were objectively used to evaluate the visual similarity of granule and solution of HLG and its placebo. The visual index results showed that the placebo had high similarity with HLG (Figures 2–4 and Table 4), and the similarity about the RGB and HSV values of granule and solution between HLG and the placebo was 1.0000, 0.9980, 0.9973, and 0.9916, respectively. The color depth similarity of solution between the granule and placebo was 0.999 (Table 5).

### 3.3. Similarity Evaluation of Taste Attributes

#### 3.3.1. Sensor Response Analysis

**Figure 5:**
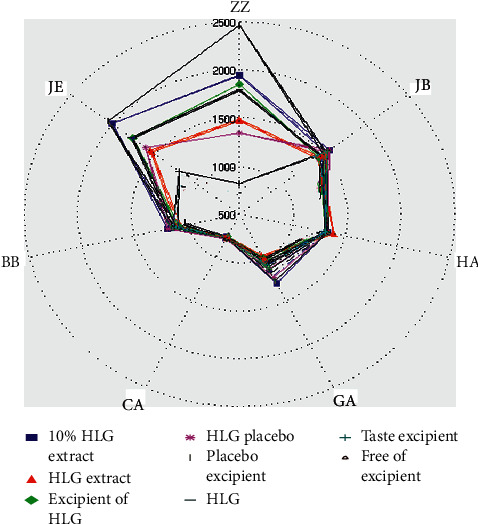
E-tongue radar chart of 7 different samples.

Figure 5 shows the radar plots of all the sensors' maximum response points for the seven samples about HLG and its placebo. As shown in Figure 5, the sensors response intensity for the taste of seven samples ranges from 500 to 2500. All the sensors used on E-tongue are cross-selective and are used to discriminate among samples. In addition, the study theorized that using all of the sensors could discriminate these samples at lower concentrations because differences at the lower concentrations may be less apparent and more difficult to detect [37]. Furthermore, the higher response intensity represents the better taste correlation (or more similarity) between different samples or groups [33]. In particular, the response intensity of HLG and its placebo on the three sensors ZZ, JB, and JE was comparable, and the response intensity was about 2000, indicating that the sensors ZZ, JB, and JE had a clear discrimination. Three of other sensors (HA, GA, and BB) had a response value of around 1500, and a low response value was recorded for the CA sensor. The response intensity of HLG on the six sensors ZZ, JE, BB, CA, JA, and JB was stronger than that of the placebo. The response intensity of the placebo on the HA sensor was slightly greater than that of HLG, but the difference was not significant. All in all, the data of E-tongue showed that the taste of the placebo was close to that of HLG, and they had good similarity.

#### 3.3.2. PCA Analysis

**Figure 6:**
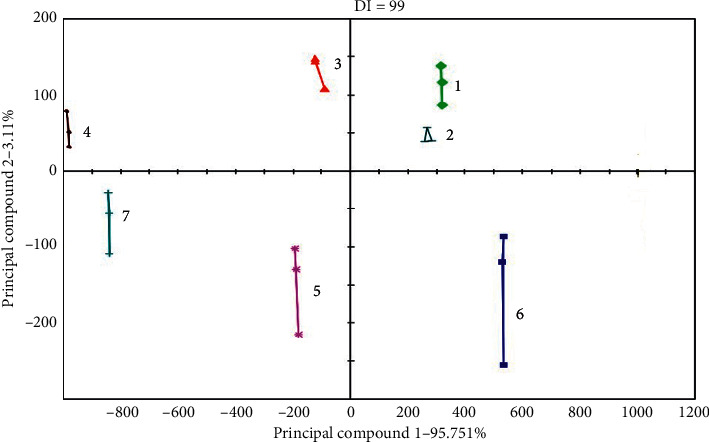
The PCA map of E-tongue of HLG and the placebo (1, HLG extract; 2, HLG; 3, excipient of HLG; 4, free of excipient in placebo; 5, HLG placebo; 6, 10% HLG extract; 7, taste excipient of HLG placebo.

**Figure 7:**
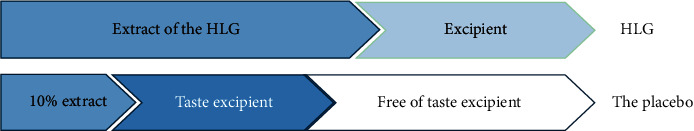
The diagram of the composition of HLG and the placebo.

According to the signals from E-tongue, the signal values were used as the inputs of PCA.  Figure 6 shows that the total contribution rate of PC1 and PC2 reached 97.5%. The information of the two principal components could represent the 97.5% response values of E-tongue for 7 samples, and the DI was 99, so that the samples could be effectively distinguished from each other. In Figures 6 and 7, HLG was composed of 100% extract and excipient. The taste source of HLG was totally from the HLG extract. Therefore, the excipient was far away from the granule on the PCA map. The placebo consisted of 10% extract, taste excipient, and free of excipient. The 10% extract, taste excipient, and placebo were close to each other on PCA map, and free of excipient was far away from the placebo. As shown in the PCA chart, the distance between HLG and the placebo was relatively closer in the direction of PC1. The taste of HLG and the placebo was considered to be similar, but there were few differences.

#### 3.3.3. The Taste Similarity of HLG and the Placebo

According to the above experiment methods, three batches of HLG and the placebo were determined in the E-tongue study. The response values on the seven sensors were recorded, and the similarity was calculated by the angle cosine algorithm. The results showed that the taste between HLG and its placebo was similar to 0.8616 (Table 6).

### 3.4. Human Sensory Evaluation

Generally accepted evaluation methods in the world were mainly subjective evaluation.  Table 7 provides the sensory evaluation results of HLG and the placebo. The significance level *P* for parameters (appearance, color, and clarity) was >0.05, representing the significant difference between smell and taste. As can be seen, the overall similarity of the two preparations was 0.9827, which was considered to be similar. Hence, the correlation of the established objective evaluation methods and the subjective evaluation methods was expressed by similarity, which was represented by the angle cosine values.

### 3.5. Objective and Human Sensory Evaluation Results

The purpose of this study was to develop methods for the similarity evaluation between the placebo and test preparation. For this purpose, the human sensory evaluation methods by scoring combined with the intelligent methods based on the CQAs were applied in the similarity evaluation of HLG and its placebo. Owing to that, the intelligent evaluation results and human sensory evaluation results were close to 1 (Table 8), and the established intelligent evaluation methods were reliable and effective.

## 4. Discussions

It is reported that detection of critical quality attributes is the premise of granule quality control [38], and it is the main aspect leading to the similarity of placebo granules. Each of sensory characteristic has been always a challenge mimicking TCM placebo. So, in this research, CQA was applied to similarity evaluation of HLG and its placebo for achieve quality control.

As a special preparation, the placebo also must meet CQAs of conventional granule. Formulation attributes were used for quantitation of CQAs of solid oral dosage more than once, which play an important role during the pharmaceutical manufacturing process [39]. In this study, to ensure the stability and uniformity of the two preparations, the physical properties of the preparations including angle of repose, moisture content, the median particle size, dissolubility, density, viscosity, and pH value were used as the key indexes of the formulation attributes to characterize the degree of similarity between the tested preparation and placebo about granule and solution by the angle cosine values. Some general reports showed that the physical properties were evaluated as the quality of the traditional dosage forms [28] and the particles formability and solubility. Investigating the physical properties of granule formability and solubility will help in evaluating the sensory characteristic of granule and solution. Recently, few studies proposed that the physical properties were characterized as granule sensory characteristic that people can perceive by the eyes and hands. The evaluation of CQAs of granule at the different states is beneficial to the preparation of the placebo. According to dissolubility, density, and viscosity of solution, appropriate excipient is used for placebo simulation. For instance, the clarity was as a kind of visual information and difficult to be objectively described, which was characterized as solution solubility. The dissolubility, density, viscosity, and pH have been shown to be strongly related to the clarity of the solution. Thus, those correlated physical properties were influence factors that evaluated HLG formulation attributes. However, simple numerical comparisons cannot be used to fully characterize granule similarity. The Euclidean distance and the angle cosine usually measured similarity, and the angle cosine value was adopted to compare similarities in this study. According to the limitations of Euclidean distance as the measure between points similarity, the chaotic local weighted linear forecast algorithm based on the angle cosine is proposed, which replaces Euclidean distance by cosine in the measurement of the similarity between phase points [40]. More and more researchers performed chromatographic fingerprint similarity analysis by the angle cosine, owing to overcoming the disadvantages of the chaotic local prediction algorithm based on the Euclidean distance. Therefore, an intuitive and practical way to assessment similarity of granule based on the angle cosine was developed [41]. From the above results, the TCM placebo had high similarity with HLG in the dosage form attributes. The monitoring of granule formulation attributes is conducive to the validation and amplification of the next preparation process of placebo.

As one of the most important indices, the visual attribute is a difficulty for mimicking TCM placebo. The world of human vision is colorful and that is a salient aspect of visual information [40]. Generally, the visual feature attributes of TCM placebo were determined through sensory and subjective determination, which could lead to biased assessments and lack of objectivity. Although performing the clinical trials remains subjective and the studies on objective assessment methods for placebo are lacking, the visual assessment methods for placebo is essential [42]. Therefore, some researchers found that color card and the computer vision system has begun to be applied in relevant medicine research studies. For example, gray reference card was recorded as tooth color to standardize color assessment in dental photography [42]. Stool color card was screened for early detection of biliary atresia and long-term native liver survival in Japan [43]. As a consequence, owing to the sensitivity and specificity of granule visual information, the color card as a reference was used to evaluate the color difference of granule and solution of two preparations in this work. The closer the card number, the more similar the color of the two preparations was. Compared with conventional scoring sensory evaluation, color card evaluation can more accurately represent the difference and degree of color, and it is an effective evaluation method that combined subjective evaluation with objective evaluation [44].

Additionally, the study discussed and provided some recent technologies on how the computer vision system, combined with image capturing and data analysis, could lead to the digitization of visual feature attributes for assessing the visual similarity between the HLG and its placebo, while it also provided objective data for placebo preparation. Computer vision system is a rapid, nondestructive, nonexpansive, efficient, repeatable, precise, and consistent technique, and it has been used for color analysis of the sensorial attributes of herb medicine, such as different groups of Semen Arecae [10]. Research had been focused on developing intelligent sensory technologies for evaluation of the similarity between the HLG and its placebo.

The human visual information of HLG and its placebo was not easy to accurately be described, so this study developed a novel computer vision system based on human visual information, which was used to satisfy digital of vision. Recently, image processing and machine learning techniques have been combined to develop a CV system whose configuration and tuning have been strongly simplified that makes easier its deployment in real applications [45]. The CV system as a novel method was discriminated Semen Arecae and its processed products through analyzing RGB of color parameters [10]. In addition to the color, the CV system was other visual experiences that included the HSV color space and gray value. Pothen and Nuske proposed a vision-based system to evaluate the ripeness of grapes using the H component of the hue saturation value (HSV) color space [46]. In Rahman and Hellicar, RGB and HSV color features extraction and SVM classifiers were training to predict mature grape bunches and undeveloped grape bunches [47]. The RGB and HSV values were different in visual representations, and the gray values were prescribed as the color depth of each point of image. The RGB values were used to accurately record the image color by the CV system, and the HSV values were used to describe the visual perception face to the user. In this study, the color of the sample images was described and compared from three aspects, and the results indicated that the similarity between the HLG granule and placebo was quite close, no matter from RGB color space, HSV space, or image gray value. The visual index results all showed that the visual attributes of HLG were highly similar to those of the placebo.

During the clinical trials, the simplest taste evaluation method for TCM placebo and test preparation was to provide a measure of taste through human perception. Therefore, there were no regulatory-defined parameters to evaluate similarity of samples, which resulted in a lack of objective taste evaluation methodologies. However, the E-tongue may be a better alternative, as it could profile the taste attributes of the preparation via an array of sensors. Compared with human perception, the E-tongue could also provide a more objective and consistent taste evaluation without limits. The difference in sensor response patterns is the key to discriminate the HLG and its placebo in this research [10].

Some studies exhibit potential advantages, and a concise objective taste assessment tool using the electronic tongue in the assessment of DEH taste attributes in the food industry. The E-tongue has been studied as an objective taste assessment tool for the qualitative and quantitative characterization of peanut meal enzymatic-hydrolysis hydrolysates [23]. Thus, the E-tongue conducted objective taste evaluation in this investigation and comparison of taste differences between two preparations with the help of electronic tongue. The taste evaluation between placebo and HLG mimic the human tongue to sense the different tastes by the low-selectivity taste sensors of E-tongue. The sensory response signals combined with the multivariate data analysis system can help characterizing taste of solution. According to the sensory response signals obtained from E-tongue, the taste similarity of two preparations was represented by the angle cosine, and it can represent different tastes.

The main purpose of research was its use in mimicking placebo that aimed to assess the similarity of placebo. Thus, we showed that the similarity results of the human sensory evaluation method and the intelligent methods have no significantly differences.

## 5. Conclusions

Although conventional methods of sensory analysis such as human sensory evaluation are currently used for checking quality and sensory similarity, they may not be the most appropriate evaluation techniques for the test preparation and placebo used for clinical trials. Despite obvious challenges, we prepared a HLG placebo formulation that was well matched to HLG in terms of taste, smell, and appearance at the granule and solution status. To evaluate the similarity of HLG and its placebo, we developed a comprehensive sensory similarity evaluation method that included visual information and taste information, which was characterized by means of the CV system and E-tongue. The formulation attributes were quantified by physical property testers. Furthermore, we concluded that this well-characterized granule and solution could be used as a placebo to facilitate controlled human intervention studies assessing the similarity to the test preparation.

For the specialty of evaluation indexes and TCM placebo, applications of the comprehensive similarity evaluation method in the field of sensory evaluation are novel. In addition, the angle cosine values were used as indicators to evaluate the similarity and characterize the correlation between human sensory evaluation methods and intelligent sensory evaluation methods. This study highlighted the important role of this similarity evaluation indicator (angle cosine values) for exploring intelligent sensory evaluation technologies and human sensory evaluation methods based on CQAs for novel applications in pharmacology.

For the CV system to be meaningful in visual information digitization, extracting RGB, HSV, and gray values of samples is very important. These parameters should be taken as a color reference for the simulation of the test preparation. For E-tongue to be useful in taste evaluation of samples and choice of taste standards, the types of samples and their concentrations are very important. Different from human sensory evaluation, E-tongue reflects the macroscopic sensory taste characteristics of samples and can be successfully used to screen for better taste agents and for optimal concentrations. It would have the benefit to provide pharmaceutical researchers with reliable data quickly concerning the taste of a product and without the need for testing a product on human volunteers. Although perception of samples is partially subjective, with further research, human sensory evaluation methods and intelligent sensory evaluation methods could be used in clinical situations to assess improvements in drug sensory quality control and medication compliance, while helping to avoid the objective system causing errors.

## Figures and Tables

**Table 1:** Formulation attributes of HLG and the placebo.

Index	HLG 160301	Placebo18041801	Angle cosine values
Angle of repose (°)	38.00 ± 1.00	38.83 ± 1.26	0.9991
Moisture (%)	2.21 ± 0.12	2.36 ± 0.16	0.9952
Median particle size (*μ*m)	642.20 ± 5.31	656.97 ± 22.18	0.9986
Dissolubility	Basically dissolved, a small amount of precipitate at the bottom	Basically dissolved, a small amount of precipitate at the bottom	
Similarity		0.9976	

Data represent means ± standard deviation (*n* = 3 for formulation attributes).

**Table 2:** Similarity of the solution clarity between HLG and the placebo.

Sample name	Density (g/cm^3^)	Viscosity (cp)	Dissolubility (%)	pH	Cosine
HLG solution	1.02 ± 0.00	1.87 ± 0.06	0.97 ± 0.02	4.65 ± 0.01	0.9997
Placebo	1.02 ± 0.01	1.80 ± 0.00	0.94 ± 0.02	4.66 ± 0.02	

Data represent mean ± standard deviation (*n* = 3 for formulation attributes).

**Table 3:** The color card evaluation results between HLG and the placebo.

Sample name	Color card number	Sample name	Color card number
HLG	2310C	HLG solution	1605C
Placebo	2312C	Placebo solution	1603C

*n* = 6 for sensory evaluation scores.

**Table 4:** Similarity of visual indicators of the granule and solution between HLG and the placebo.

Visual index	Granule		Solution	
	RGB	HSV	RGB	HSV
Similarity (cosine)	1.0000	0.9980	0.9973	0.9916

**Table 5:** The results of gray value measurement between HLG and the placebo at solution status.

Number	Sample name	Pixel mean (SRM)
1	Placebo 18041801	64.94 ± 1.09
2	HLG 160301	70.37 ± 0.33
Similarity (cosine)	0.9999	

Data represent means ± standard deviation (*n* ＝ 3 for gray value).

**Table 6:** The results of sensor measurement on HLG and the placebo.

Sensor	HLG	Placebo
ZZ	2098.51	2052.12
JE	1976.81	1941.32
BB	1712.37	1633.02
CA	923.34	922.85
GA	1584.43	1466.12
HA	1431.92	1501.04
JB	2022.90	1965.45
Cosine	0.8616	

**Table 7:** Human sensory evaluation similarity results between HLG and the placebo.

Indexes	HLG	Placebo	Cosine	*P*
Appearance	3.67 ± 0.26	3.25 ± 0.27	0.9998	0.175
Color	3.75 ± 0.27	3.42 ± 0.38	0.9992	0.175
Clarity	3.42 ± 0.38	3.50 ± 0.32	0.9835	0.594
Smell	3.67 ± 0.26	2.58 ± 0.74	0.9998	0.001
Taste	3.67 ± 0.26	2.87 ± 0.54	0.9798	0.003
Similarity (cosine)	0.9827			

Data represent means ± standard deviation (*n* ＝ 6 for sensor scores).

**Table 8:** Statistics on the similarity evaluation results of the HLG placebo.

Evaluation project	Index	Evaluation methods	Results/Similarity (cosine)
Formulation attributes	The median particle size	Laser particle size analyzer	0.9986
	Moisture	Rapid moisture analyzer	0.9952
	Dissolubility	Stir and dissolve	Basic dissolution
	Fluidity	Powder comprehensive characteristic tester	0.9991
	Clarity	Density, viscosity, dissolubility, and pH	0.9835
Visual feature attributes	Visual	Sensory evaluation	0.9947
		Color card	Color card number is similar
		RGB space (granule/solution)	1.000/0.9973
		HSV space (granule/solution)	0.9980/0.9916
		Gravy value (granule)	0.9999
		Clarity (sensory evaluation)	0.9997
Smell and taste attributes	Smell	Sensory evaluation	0.9993
	Taste	Sensory evaluation/electronic tongue	0.9999/0.8616

## Data Availability

The data used to support the findings of this study are included within the supplementary information files.

## Supplementary Materials

Supplementary Materials The data 1 are the formulation attributes raw data of HLG and its placebo (Tables 1-2 and Figure 1). The data 2 are the color card raw results of granule between HLG and its placebo (Table 3). The data 3 are the computer vision system raw results of granule and solution between HLG and its placebo (Tables 4-5 and Figures 2–4). The data 4 are the human sensory evaluation raw results of granule and solution between HLG and its placebo (Table 8).

## References

[1] Zhang W. G. (2018). *Nearly A Decade of Research and Development of New Drug of TCM Literature Metrology Analysis*.

[2] Zhong Y.-Q., Fu J.-J., Liu X.-M. (2010). The reporting quality, scientific rigor, and ethics of randomized placebo-controlled trials of traditional Chinese medicine compound formulations and the differences between Chinese and non-Chinese trials. *Current Therapeutic Research*.

[3] Jütte R. (2013). The early history of the placebo. *Complementary Therapies in Medicine*.

[4] Wu F., Wang Y. J., Hong Y. L. (2012). Research progress in preparation and clinical use of traditional Chinese medicine placebo. *Chinese Journal of New Drugs*.

[5] Zhou Q., Wang Y., Zhang J. (2018). Fingerprint analysis of Huolingshengji Formula and its neuroprotective effects in SOD1G93A mouse model of amyotrophic lateral sclerosis. *Scientific Reports*.

[6] Wang L., Niu Q., Hui Y., Jin H., Chen S. (2015). Assessment of taste attributes of peanut meal enzymatic-hydrolysis hydrolysates using an electronic tongue. *Sensors*.

[7] Pavlou A. K., Magan N., Mcnulty C. (2002). Use of an electronic nose system for diagnoses of urinary tract infections. *Biosensors and Bioelectronics*.

[8] Wojnowski W., Dymerski T., Gbicki J. (2019). Electronic noses in medical diagnostics. *Current Medicinal Chemistry*.

[9] D’Amico, Arnaldo, Pennazza G., Santonico M. (2010). An investigation on electronic nose diagnosis of lung cancer. *Lung Cancer*.

[10] Xu M., Yang S.-L., Peng W. (2015). A novel method for the discrimination of semen arecae and its processed products by using computer vision, electronic nose, and electronic tongue. *Evidence-Based Complementary and Alternative Medicine*.

[11] Fitriyono A., Vincenzo F., Ruud V. (2021). Surface color distribution analysis by computer vision compared to sensory testing: vacuum fried fruits as a case study. *Food Research International*.

[12] Wasilewski T., Migoń D., Gębicki J., Kamysz W. (2019). Critical review of electronic nose and tongue instruments prospects in pharmaceutical analysis. *Analytica Chimica Acta*.

[13] Breitkreutz J., Boos J. (2007). Paediatric and geriatric drug delivery. *Expert Opinion on Drug Delivery*.

[14] Haraguchi T., Yoshida M., Kojima H., Uchida T. (2016). Usefulness and limitations of taste sensors in the evaluation of palatability and taste-masking in oral dosage forms. *Asian Journal of Pharmaceutical Sciences*.

[15] Zidan A. S. (2017). Taste-masked tacrolimus-phospholipid nanodispersions: dissolution enhancement, taste masking and reduced gastric complications. *Pharmaceutical Development and Technology*.

[16] Chikukwa M. T. R., Wesoly M., Korzeniowska A. B., Ciosek-Skibinska P., Walker R. B., Khamanga S. M. M. (2020). Assessment of taste masking of captopril by ion-exchange resins using electronic gustatory system. *Pharmaceutical Development and Technology*.

[17] Kiani S., Minaei S., Ghasemi-Varnamkhasti M. (2017). Integration of computer vision and electronic nose as non-destructive systems for saffron adulteration detection. *Computers and Electronics in Agriculture*.

[18] Amann A., Poupart G., Telser S., Ledochowski M., Schmid A., Mechtcheriakov S. (2004). Applications of breath gas analysis in medicine. *International Journal of Mass Spectrometry*.

[19] Preis M., Eckert C., Häusler O., Breitkreutz J. (2014). A comparative study on solubilizing and taste-masking capacities of hydroxypropyl-*β*-cyclodextrin and maltodextrins with high amylose content. *Sensors and Actuators B: Chemical*.

[20] Peng J. H., Xue Z. F., Zhang L. N. (2018). Moisture sorption and diffusion determination of Chinese herbal granule: moisture-resistant effects of fluidized bed granulation with dextrin. *Chinese Herbal Medicines*.

[21] Lin Z., Zhang Q., Liu R. (2016). Evaluation of the bitterness of traditional Chinese medicines using an E-tongue coupled with a robust partial least squares regression method. *Sensors*.

[22] Wu S. p., Xu L. p., Guo Y. (2015). Application status of placebo in clinical research in China for the last 30 years. *Chinese Journal of Traditional Chinese Medicine*.

[23] Wang C., Huang H., Jia M., Jin S., Zhao W., Cha R. (2015). Formulation and evaluation of nanocrystalline cellulose as a potential disintegrant. *Carbohydrate Polymers*.

[24] Phetpan K., Udompetaikul V., Sirisomboon P. (2019). In-line near infrared spectroscopy for the prediction of moisture content in the tapioca starch drying process. *Powder Technology*.

[25] Murav A. V., Zaitsev L. G., Murav A. A. (2000). Microheological properties of different erythrocyte populations in persons with elevated arterial pressure and in physically active persons. *Human Physiology*.

[26] Guan X., Liu J., Huang K., Kuang J., Liu D. (2019). Evaluation of moisture content in processed apple chips using nirs and wavelength selection techniques. *Infrared Physics & Technology*.

[27] Reynolds J. G., Mauss B. M., Daniel R. C. (2018). The relative viscosity of NaNO_3_ and NaNO_2_ aqueous solutions. *Journal of Molecular Liquids*.

[28] Mian X., Ling J., Guanqin W. (2012). Chaotic local weighted linear prediction algorithms based on the angle cosine. *Systems Engineering Procedia*.

[29] Li Q., Huang Q., Fu X. J. (2018). Application of color quantitative analysis in quality evaluation of rhubarb charcoa. *Chinese Journal of Experimental Traditional Medical Formulae*.

[30] Zhao C. Y. (2018). Principle and implementation of digital image processing sharpening technology. *Electronic Technology and Software Engineering*.

[31] Song Y. S., Wang X. N., Qin M. (2017). Study on enzymatic hydrolysis of grass carp fish skin and promotion of Streptococcus thermopilus proliferation by the hydrolysates. *Science Technology & Engineering*.

[32] Du R. C., Wang Y. J., Wu F. (2013). Distinguishing the taste of traditional Chinese medicine by electronic tongue. *Chinese Journal of Traditional Chinese Medicine*.

[33] Wang Y., Feng Y., Wu Y., Liang S., Xu D. (2013). Sensory evaluation of the taste of berberine hydrochloride using an Electronic Tongue. *Fitoterapia*.

[34] Ernst G., Bosio M., Salvado A. (2015). Comparative study between sequential automatic and manual home respiratory polygraphy scoring using a three-channel device: impact of the manual editing of events to identify severe obstructive sleep apnea. *Sleep Disorders*.

[35] Bari N. K., Fazil M., Hassan M. Q. (2015). Brain delivery of buspirone hydrochloride chitosan nanoparticles for the treatment of general anxiety disorder. *International Journal of Biological Macromolecules*.

[36] Fan L. J., Fu S., Lin L. F. (2017). Application of computer color matching technique in color simulation of ruyi golden powder placebo. *Chinese Traditional and Herbal*.

[37] Schlossareck C., Ross C. F. (2019). Electronic tongue and consumer sensory evaluation of spicy paneer cheese. *Journal of Food Science*.

[38] Ma L., Li Y., Lei L. (2020). Real-time process quality control of ramulus cinnamomi by critical quality attribute using microscale thermophoresis and on-line NIR. *Spectrochimica Acta Part A: Molecular and Biomolecular Spectroscopy*.

[39] Metta N., Verstraeten M., Ghijs M., Kumar A., Schafer (2018). Model development and prediction of particle size distribution, density and friability of a comilling operation in a continuous pharmaceutical manufacturing process. *International Journal of Pharmaceutics*.

[40] Lee G. J., Lee J. H., Park J. H. (2018). Assessment of chemical equivalence in herbal materials using chromatographic fingerprints by combination of three similarity indices and three-dimensional kernel density estimation. *Analytica Chimica Acta*.

[41] Lindsey D. T., Brown A. M., Reijnen E., Rich A. N., Kuzmova Y. I., Wolfe J. M. (2010). Color channels, not color appearance or color categories, guide visual search for desaturated color targets. *Psychological Science*.

[42] Sampaio C. S., Atria P. J., Hirata R., Jorquera G. (2019). Variability of color matching with different digital photography techniques and a gray reference card. *The Journal of Prosthetic Dentistry*.

[43] Gu Y. H., Yokoyama K., Mizuta K. (2019). Stool color card screening for early detection of biliary atresia and long-term native liver survival: a 19-year cohort study in Japan. *The Journal of Pediatrics*.

[44] Yun F. W., Xin M. Y., Wu H. L. (2011). Study on preparation method and effect evaluation of Chinese medicine placebo in large double-blind clinical trial. *Traditional Chinese Drug Research & Clinical Pharmacology*.

[45] Cavallo D. P., Cefola M., Pace B., Logrieco A. F., Attolico G. (2019). Non-destructive and contactless quality evaluation of table grapes by a computer vision system. *Computers and Electronics in Agriculture*.

[46] Lichter A. (2016). Rachis browning in tablegrapes. *Australian Journal of Grape and Wine Research*.

[47] Rahman A., Hellicar A. Identification of mature grape bunches using image processing and computational intelligence methods.

