# Supplementary material for: Similarity Evaluation on the Compound TCM Formulation “Huoling Shengji Granule” and Its Placebo by Intelligent Sensory Evaluation Technologies and the Human Sensory Evaluation Method Based on Critical Quality Attributes
Source: Evid Based Complement Alternat Med. 2021 Apr 14;2021:6637326. doi: 10.1155/2021/6637326 (PMC8062196; doi:10.1155/2021/6637326)
Supplement: Supplementary Materials — The data 1 are the formulation attributes raw data of HLG and its placebo (Tables 1-2 and Figure 1). The data 2 are the color card raw results of granule between HLG and its placebo (Table 3). The data 3 are the computer vision system raw results of granule and solution between HLG and its placebo (Tables 4-5 and Figures 2–4). The data 4 are the human sensory evaluation raw results of granule and solution between HLG and its placebo (Table 8). [file 6637326.f1.zip › 6637326.f1/data(1).pdf]

	Placebo	Placebo	Placebo	means ± sta	HLG soluti	HLG soluti	HLG soluti	means ± sta
density	1.0206	1.0118	1.021	1.02±0.01	1.019	1.0189	1.0238	1.02±0
viscosity	1.8	1.8	1.8	1.8±0	1.92	1.8	1.9	1.87±0.06
Dissolubilit	0.92	0.95	0.95	0.94±0.02	0.99	0.97	0.95	0.97±0.02
PH	4.64	4.67	4.66	4.66±0.02	4.64	4.66	4.65	4.65±0.01
