# Supplementary material for: Similarity Evaluation on the Compound TCM Formulation “Huoling Shengji Granule” and Its Placebo by Intelligent Sensory Evaluation Technologies and the Human Sensory Evaluation Method Based on Critical Quality Attributes
Source: Evid Based Complement Alternat Med. 2021 Apr 14;2021:6637326. doi: 10.1155/2021/6637326 (PMC8062196; doi:10.1155/2021/6637326)
Supplement: Supplementary Materials — The data 1 are the formulation attributes raw data of HLG and its placebo (Tables 1-2 and Figure 1). The data 2 are the color card raw results of granule between HLG and its placebo (Table 3). The data 3 are the computer vision system raw results of granule and solution between HLG and its placebo (Tables 4-5 and Figures 2–4). The data 4 are the human sensory evaluation raw results of granule and solution between HLG and its placebo (Table 8). [file 6637326.f1.zip › 6637326.f1/data(2).pdf]

	HLG	placebo	HLG soluti	placebo solution
color card r	2310C	2312C	1605C	1603C
