# Supplementary material for: Similarity Evaluation on the Compound TCM Formulation “Huoling Shengji Granule” and Its Placebo by Intelligent Sensory Evaluation Technologies and the Human Sensory Evaluation Method Based on Critical Quality Attributes
Source: Evid Based Complement Alternat Med. 2021 Apr 14;2021:6637326. doi: 10.1155/2021/6637326 (PMC8062196; doi:10.1155/2021/6637326)
Supplement: Supplementary Materials — The data 1 are the formulation attributes raw data of HLG and its placebo (Tables 1-2 and Figure 1). The data 2 are the color card raw results of granule between HLG and its placebo (Table 3). The data 3 are the computer vision system raw results of granule and solution between HLG and its placebo (Tables 4-5 and Figures 2–4). The data 4 are the human sensory evaluation raw results of granule and solution between HLG and its placebo (Table 8). [file 6637326.f1.zip › 6637326.f1/data(3).pdf]

rs of the Huoling shengji Granules and Placebo

Visual indi	HLG Granules 160301			placebo 18041801
R means	76.0065	64.0331	69.8235	65.6489
G means	62.8133	52.4682	57.9655	54.4878
B means	47.8231	40.4417	42.7747	40.5399
R variance	191.3295	136.3164	170.3477	154.5817
G variance	180.3956	129.0387	158.0126	149.4524
B variance	167.7381	121.7773	143.5658	139.6748
R standard	13.8322	11.6755	13.0517	12.4331
G standard	13.4311	11.3595	12.5703	12.2251
B standard	12.9514	11.03527	11.9819	11.8184
similarity	1			

The results of HSV visual indicators of th

Visual indi	HLG Granules 160301			placebo 18041801
H means	0.0885	0.0847	0.0936	0.0923
S means	0.3819	0.3796	0.3991	0.3951
V means	0.2981	0.2511	0.2738	0.2574
H variance	9.33E-05	9.80E-05	0.0001	0.0001
S variance	0.0057	0.0058	0.0062	0.0066
V variance	0.0029	0.0021	0.0026	0.0021
H standard	0.0097	0.0099	0.0108	0.0115
S standard (	0.0754	0.076	0.0785	0.081
V standard	0.0542	0.0458	0.0512	0.0488
similarity	0.998			

The Results of gray value color difference measurement of the Huoling shengji Granules and Placebo

Number	Sample nan	Pixel mean （SRM）
1	placebo 18	63.6911
2	placebo 18	65.4073
3	placebo 18	65.7234
4	the Huoling	70.0089
5	the Huoling	70.4311
6	the Huoling	70.6591
similarity	0.9999	

rs of the Huoling shengji Granules and Placebo

Visual indi	HLG solution 160301			placebo solution 18041801
R means	13.7884	13.9713	20.5576	16.5445
G means	11.1897	9.7496	16.3059	14.4673
B means	11.7198	10.4392	16.45	13.7709
R variance	4.9177	2.0241	3.402	2.0422
G variance	2.8295	1.6387	3.5944	1.9044
B variance	2.2902	1.6009	4.8972	2.1784
R standard	2.2176	1.4227	1.8445	1.4291
G standard	1.6821	1.2801	1.8959	1.38
B standard	1.5133	1.2653	2.213	1.4759
similarity	0.9973			

The results of HSV visual indicators of th

Visual indi	HLG solution 160301			placebo solution 18041801
H means	0.7255	0.6504	0.4941	0.3938
S means	0.2027	0.1549	0.2271	0.1884
V means	0.0541	0.0451	0.0806	0.0649
H variance	0.1341	0.1615	0.217	0.1635
S variance	0.0045	0.0084	0.0016	0.0032
V variance	7.52E-05	3.95E-05	5.24E-05	3.14E-05
H standard	0.3662	0.4019	0.4658	0.4043
S standard (	0.0672	0.0917	0.2271	0.0566

V standard	0.0087	0.0063	0.0072		0.0056
similarity	0.9916				
