# Supplementary material for: Similarity Evaluation on the Compound TCM Formulation “Huoling Shengji Granule” and Its Placebo by Intelligent Sensory Evaluation Technologies and the Human Sensory Evaluation Method Based on Critical Quality Attributes
Source: Evid Based Complement Alternat Med. 2021 Apr 14;2021:6637326. doi: 10.1155/2021/6637326 (PMC8062196; doi:10.1155/2021/6637326)
Supplement: Supplementary Materials — The data 1 are the formulation attributes raw data of HLG and its placebo (Tables 1-2 and Figure 1). The data 2 are the color card raw results of granule between HLG and its placebo (Table 3). The data 3 are the computer vision system raw results of granule and solution between HLG and its placebo (Tables 4-5 and Figures 2–4). The data 4 are the human sensory evaluation raw results of granule and solution between HLG and its placebo (Table 8). [file 6637326.f1.zip › 6637326.f1/data(4).pdf]

smell

	HLG	placebo
	3.5	3
	4	2.5
	3.5	3
	4	3.5
	3.5	2
	3.5	1.5
	3.67±0.26	2.58±0.74

appearance

	HLG	placebo
	3.5	3.5
	3.5	3.5
	4	3
	3.5	3
	3.5	3
	4	3.5
	3.67±0.26	3.25±0.27

color

clarity

	HLG	placebo
	4	3.5
	3.5	3.5
	3.5	3
	3	4
	3.5	3.5
	3	3.5
	3.42±0.38	3.5±0.32

taste

	HLG	placebo
	3.5	3
	3.5	2.5
	4	2
	4	3
	3.5	3.5
	3.5	3.2
	3.67±0.26	2.87±0.54

HLG	Placebo
3.5	4
3.5	3.5
4	3.5
4	3
3.5	3
4	3.5
3.75±0.27	3.42±0.38
